# In Vitro Safety and Efficacy Evaluation of a *Juniperus communis* Callus Culture Extract and *Matricaria recutita* Processing Waste Extract Combination as a Cosmetic Ingredient

**DOI:** 10.3390/plants13020287

**Published:** 2024-01-18

**Authors:** Anna Ramata-Stunda, Martins Boroduskis, Laura Pastare, Marta Berga, Liene Kienkas, Liene Patetko, Gundars Skudrins, Dace Reihmane, Ilva Nakurte

**Affiliations:** 1 Alternative Plants, Ltd., 2 Podraga Str., LV-1007 Riga, Latvia; martins@alternativeplants.eu (M.B.); dace@alternativeplants.eu (D.R.); 2 Institute for Environmental Solutions, “Lidlauks”, Priekuli Parish, LV-4126 Cesis, Latvia; laura.pastare@vri.lv (L.P.); marta.berga@vri.lv (M.B.); gundars.skudrins@vri.lv (G.S.); ilva.nakurte@vri.lv (I.N.); 3 Field and Forest, Ltd., 2 Izstades Str., Priekuli Parish, LV-4126 Cesis, Latvia; liene.kienkas@fieldandforest.lv; 4 Faculty of Biology, University of Lavia, 1 Jelgavas Str., LV-1004 Riga, Latvia; liene.patetko@lu.lv

**Keywords:** German chamomile, valorization, juniper, plant callus culture, chemical composition, cytotoxicity, phototoxicity, collagen production

## Abstract

For skin health promotion and cosmetic applications, combinations of plant cell extracts are extensively utilized. As most natural ingredient suppliers offer crude extracts from individual plants or specific isolated compounds, the potential interactions between them are assessed in the development phase of cosmetic products. The industry seeks extract combinations that have undergone optimization and scrutiny for their bioactivities. This study presents a combination of two sustainably produced botanical ingredients and outlines their chemical composition, in vitro safety, and bioactivity for skin health enhancement. The amalgamation comprises the extract of *Matricaria recutita* processing waste and the extract from *Juniperus communis* callus culture. Chemical analysis revealed distinct compounds within the extracts, and their combination led to a broader array of potentially synergistic compounds. In vitro assessments on skin cells demonstrated that the combination possesses robust antioxidant properties and the ability to stimulate keratinocyte proliferation, along with regulating collagen type I and matrix metalloproteinase 1 (MMP-1) production by dermal fibroblasts. The identified traits of this combination render it an appealing cosmetic component. To the best of our knowledge, this represents the first case when the extracts derived from medicinal plant processing waste and biotechnological plant cell cultivation processes have been combined and evaluated for their bioactivity.

## 1. Introduction

A growing understanding of the significance of maintaining healthy, youthful-looking skin has led to a significant increase in the demand for skincare products in recent years. Consumers now seek products that are not only effective but also safe and sustainable [1,2]. As a result, the integration of natural substances into skincare products has garnered substantial attention, offering a potential solution to address these evolving consumer preferences [3,4]. Among the vast array of natural substances available, juniper (*Juniperus communis*) and German chamomile (*Matricaria recucita*) have emerged as valuable contenders, renowned for their remarkable therapeutic properties and diverse bioactive compounds. These botanical extracts have been utilized in traditional medicine for centuries and are now being harnessed for their potential applications in skincare products [4,5,6,7].

The production of essential oils from various botanical sources is a widely practiced industry, and chamomile is one of the target plants highly valued for its therapeutic properties. During the distillation process of chamomile flowers, waste materials are generated, often referred to as chamomile herb processing waste. This waste stream, typically considered agricultural waste, presents an opportunity for valorization [8,9,10,11]. The post-distilled biomass still contains bioactive compounds that are available and sustainable for utilization in various industries, including skincare [8,9,10].

In vitro plant cell cultures involve the laboratory growth of cells derived from plant tissues. These cultures offer several advantages over traditional cultivation methods, including the ability to produce bioactive compounds in controlled environments, independent of geographical limitations and seasonal variations [12,13,14]. Juniperus, a genus of coniferous plants known for their aromatic and medicinal properties, can be harnessed through in vitro cell cultures to obtain a consistent and reliable source of bioactive compounds for skincare applications [15]. In vitro cell cultures of Juniperus provide a controlled environment that enables the production of specific bioactive compounds in higher concentrations compared to those found in the wild. This controlled cultivation allows for the optimization of growth conditions and nutrient supply, resulting in enhanced yields of desired compounds. 

Despite the positive aspects, it is essential to remember that not all natural ingredients, particularly combinations of various extracts, are inherently safe or advantageous for consumption. Certain natural compounds have the potential to induce adverse reactions or trigger allergies [12]. To ensure the safety of new skincare ingredients and enhance their market acceptability, it is advisable to conduct chemical characterization along with biological testing.

In this study, our aim was to develop a prototype skincare ingredient by combining juniper in vitro callus culture extract and an extract of chamomile essential oil processing waste. Furthermore, we conducted chemical and in vitro biological testing to assess the safety and potential efficacy of this novel blend, with the ultimate goal of facilitating the development of a promising new ingredient for skincare applications. The integration of in vitro cell cultures of Juniperus and the valorization of chamomile waste exemplified innovative approaches in the skincare industry. This study contributed to the ongoing evolution of the cosmetics industry towards greater sustainability and presented promising avenues for meeting the demands of conscious consumers seeking natural, sustainably sourced, and thoroughly in vitro-tested skincare ingredients. The in vitro evaluation of the novel ingredient in validated safety assays and bioactivity tests provided important prerequisites for further dermatological testing and product development.

## 2. Results

### 2.1. Chemical Characterization of Individual Extracts and Extract Combination

The total phenolic, flavonoid, tannin, and sugar contents were determined in both individual extracts, *M. recutita* processing by-product extract (701) and *J. communis* callus culture extract (JC), as well as in their combined form (JC + 701, 1:2). Initial screening results (Table 1) indicated that the *M. recutita* processing by-product extract (701) exhibited higher contents of these compounds (except for total tannins) compared to the *J. communis* callus culture extract. 

The most predominant group in terms of concentration (mg per 1 mL of extracts) was sugars, reaching 24.2 mg per mL in the combination extract. The next dominant groups in the combination, with similar concentrations, were total phenolics (1.6 mg per mL), total tannins (1.5 mg per mL), and total flavonoids. The latter is expressed in two ways: apigenin equivalents (APEs) and catechin equivalents (CAEs) in mg per mL extract. This decision was based on obtained data from individual compound identification using liquid chromatography–high-resolution mass spectrometry (LC-HRMS) data. The obtained values indicate that the chamomile extract (701) predominantly comprises both groups of flavonoids in similar amounts. Conversely, the juniper extract (JC) is primarily dominated by the catechin group of flavonoids. However, in the combination (JC + 701, 1:2), representatives from both groups are present at 0.8 mg and 1.3 mg per mL, respectively. Undoubtedly, the antioxidant activity of the aforementioned compound group plays a critical role in natural cosmetic ingredients [3,12]. According to the 2,2-diphenyl-1-picrylhydrazyl (DPPH) scavenging test results (Table 2), *J. communis* callus culture extract (JC) exhibited slightly stronger radical scavenging capacity (62%) compared to *M. recutita* processing waste extract (701) (58%). At the same time, when antioxidative activity is expressed in Trolox equivalents, the half-maximal inhibitory concentration (IC50) values of JC extract are higher than those of 701 and the combination, indicating a slightly lower free radical scavenging activity.

A phytochemical screening was conducted to identify and evaluate the qualitative composition of both extracts (as detailed in Table 3) and to quantitatively assess the combination (JC + 701, 1:2) using the LC-HRMS technique. The analysis revealed that both extracts in the combination contained amino acids and epigallocatechin in their composition. Amino acids and their derivatives play a significant role in improving the skin’s barrier function, hydration, pH regulation, resilience against external stressors, and wound healing acceleration [16]. *J. communis* callus culture extract (JC) was distinguished from M.recutita extract (701) by its high content of epicatechin, gallocatechin, and catechin. By combining both extracts, a higher variety of catechins in the mixture was achieved, potentially leading to higher bioactivity [17,18]. 

Apigenin 7-O-glucoside, quinic acid, coumarin derivatives, and ferulic acid glucosides were present in chamomile extract (701) but not found in JC. In addition to well-known and widely used compounds in skincare products, the examination of chamomile extract (701) unveiled the presence of fatty acyls, a crucial category of biological lipids. These compounds play a significant role in the cosmetics industry, serving as the main natural source for producing surfactants [19]. 

Exclusive compounds present in the combination (JC + 701, 1:2) are uniquely derived from *J. communis* callus culture extract (JC), such as β-thujaplicin glucosides and dihydroajaconines. *M. recutita* does not contain these compounds; β-thujaplicin and its derivatives are predominantly exclusive to the *Cupressaceae* plant family. 

### 2.2. Cytotoxicity and Phototoxicity

**Figure 1:**
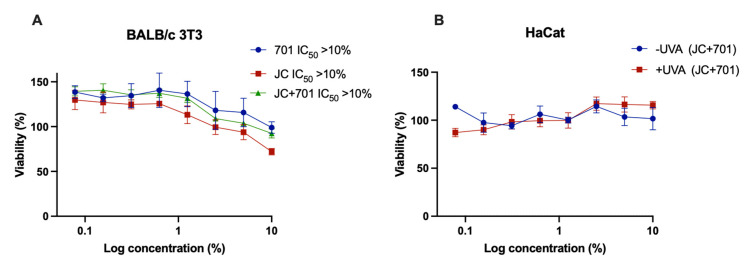
Cytotoxicity (**A**) and phototoxicity (**B**) assessment of extract combination JC + 701, 1:2. –UV: cells incubated with extracts for 1 h without UV irradiation; +UV: cells pre-incubated for 1 h with extracts and irradiated with 5 J cm^−2^ UVA; *n* = 3.

The safety of the developed extract combination was evaluated in a cytotoxicity test using embryonal fibroblast cell line BALB/c 3T3 from an albino laboratory-bred strain from a house mouse and a phototoxicity test in human keratinocyte line HaCaT. As depicted in Figure 1, no cytotoxic or phototoxic effects were observed. In fact, at the highest concentrations, a slight but variable increase in cell viability was noted. These findings support the safety of the combination, even at concentrations as high as 10% (*v*/*v*). 

### 2.3. Proliferation of Keratinocytes

**Figure 2:**
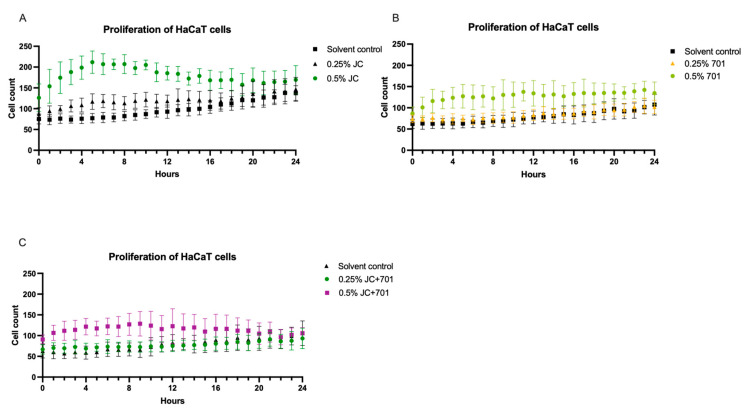
Analysis of the proliferation of HaCaT keratinocytes in the presence of JC extract (**A**), 701 extract (**B**), and combination JC + 701, 1:2 (**C**), over a 24 h period; *n* = 6.

A real-time cell monitoring system was used to assess the effect of the extract combination on the proliferation of HaCaT keratinocytes. The results indicate that the highest concentration of JC extract exhibits significant (*p* < 0.001) cell proliferation-stimulating activity compared to the control during the first 16 h after adding the extract to the cell culture. A similar, albeit less pronounced, effect was observed with the extract combination (Figure 2C), while the 701 extract did not alter the proliferative activity of HaCaT keratinocytes. 

### 2.4. In Vitro Antioxidative Activity

**Figure 3:**
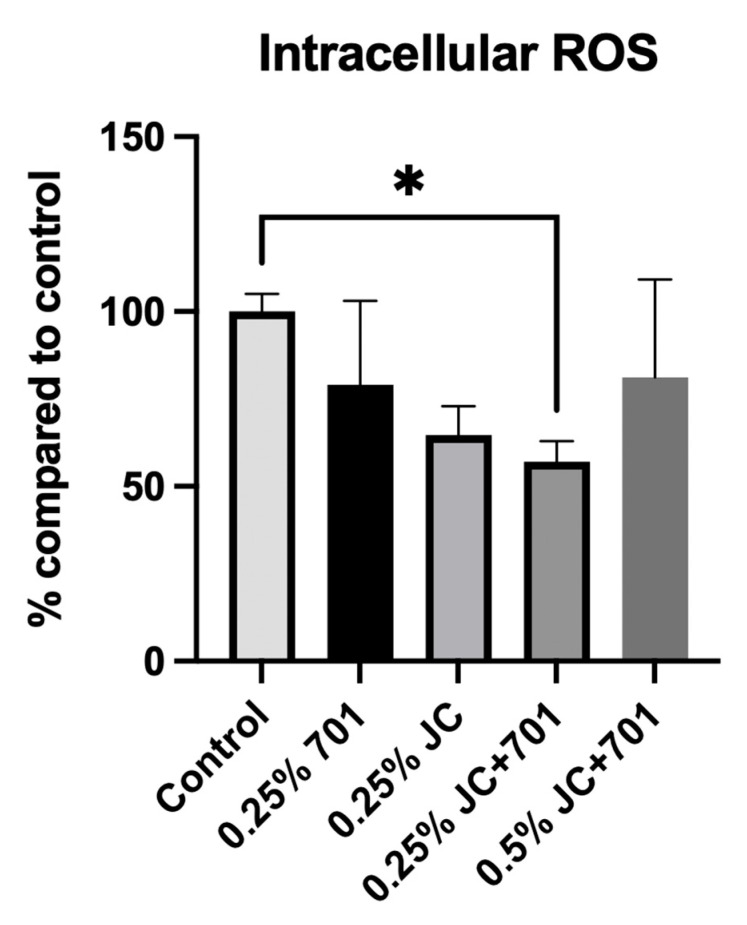
Reactive oxygen species inhibitory activity of chamomile extract (701), juniper extract (JC), and combined extract (JC + 701, 1:2) in the skin keratinocyte cell line (HaCaT). Data are expressed as a % reduction in ROS accumulation compared to the solvent control. *n* = 3. * *p* < 0.01, ANOVA.

Analysis of the extract combination’s effect on the accumulation of reactive oxygen species (ROS) in UV-irradiated HaCaT keratinocytes revealed that, while extracts JC and 701 individually influence the neutralization of free radicals, the combination exhibits the highest activity (Figure 3). When incubated with a 0.25% (*v*/*v*) extract combination, ROS levels were reduced by 42.92 ± 5.91% compared to the control. Although the majority of compounds identified in the composition are known for their antioxidative activity, their specific mechanisms of action may vary. The DPPH scavenging activities described in Section 2.1 differ from the results obtained in the flow cytometric quantification of intracellular ROS. This could be explained by extracts’ ability to both directly scavenge free radicals and promote intracellular defense mechanisms. The reduced antioxidant capacity at a concentration of 0.5% (*v*/*v*) can be attributed to cellular overstimulation or potential pro-oxidant activities of specific compounds at higher concentrations. 

### 2.5. Secretion of Pro-Collagen I Alpha 1 and Matrix Metalloproteinase 1 (MMP-1)

**Figure 4:**
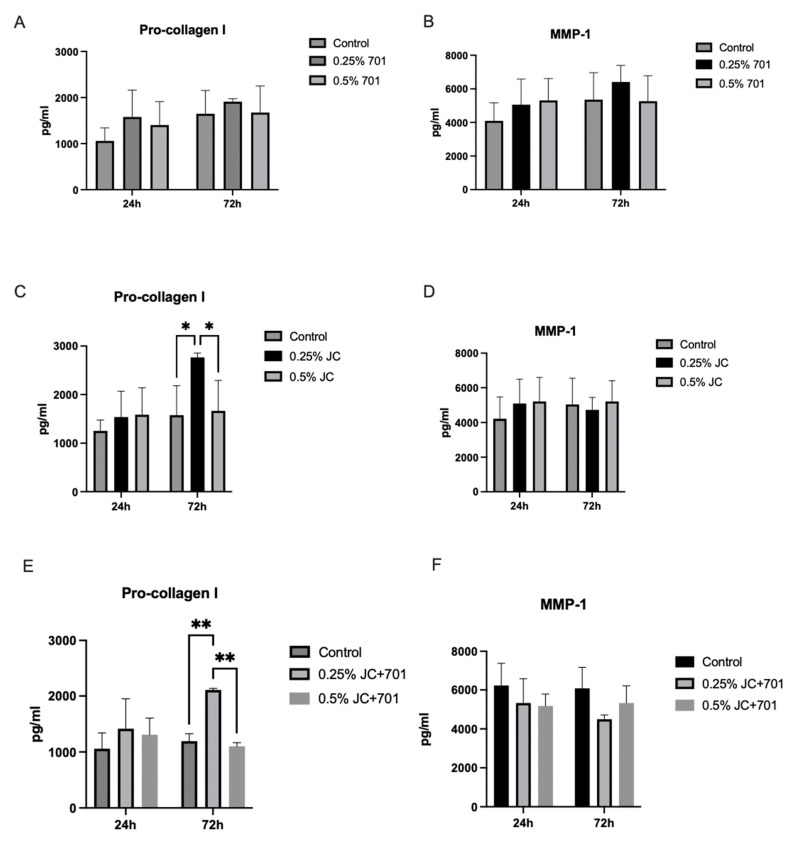
Secretion of pro-collagen I alpha 1 (**A**,**C**,**E**) and MMP-1 (**B**,**D**,**F**) in dermal fibroblasts in the presence of the extracts 701 (**A**,**B**), JC (**C**,**D**) and combination JC + 701 (**E**,**F**), 1:2. *n* = 4; * *p* < 0.05; ** *p* < 0.001, ANOVA.

Pro-collagen I alpha 1 and MMP-1 were quantified in dermal fibroblast cell cultures after 24 and 72 h of incubation with the extract combination (Figure 4). The results indicate that within the first 24 h of incubation, there was a slight increase in collagen production in the presence of individual extracts and the combination at a concentration of 0.25%; however, there was high variability among replicates. A slight decrease in MMP-1 production was observed in the presence of the combination, while extracts individually slightly increased secretion. After 72 h of incubation, a significant increase in pro-collagen I concentrations was observed in the presence of the JC extract and extract combination at 0.25% concentration; extract 701 had minimal effect. After 72 h, the pro-collagen concentration was 2767.23 ± 88.42 pg/mL for 0.25% JC and 1574.27 ± 223.17 pg/mL for the corresponding control, yielding a 75.78% increase of pro-collagen. In the case of the combination, the pro-collagen concentration in the presence of a 0.25% concentration was 2108.98 ± 28.22 pg/mL, and in the corresponding controls, 1194.02 ± 132.15 pg/mL, yielding a 76.63% increase. The average increase in the pro-collagen concentration in pg/mL compared to controls was 1192.96 pg/mL in the case of JC and 914.97 pg/mL in the case of the combination. Results allow us to conclude that JC in the combination, specifically, contributes to the pro-collagen synthesis stimulating activity. Reduced levels of MMP-1 were noted in cell cultures treated for 72 h with 0.25% of the extract combination. Individual extracts did not have MMP-1-reducing activity; 0.25% of extract 701 even slightly increased it. These data suggest that the combination stimulates collagen synthesis and exhibits slightly inhibiting activity against collagen-degrading MMP-1. 

## 3. Discussion

The results of this study demonstrate that the combination of *Matricaria recutita* processing by-product extract (701) and *Juniperus communis* callus culture extract (JC), as well as individual extracts, exhibit promising properties for potential skincare applications. The combination contains a rich array of bioactive compounds, including sugars, flavonoids, tannins, amino acids, epigallocatechin, apigenin 7-O-glucoside, quinic acid, coumarin derivatives, ferulic acid glucosides, β-thujaplicin glycosides, dihydroajaconines, and fatty acyls. These compounds have been shown to have various beneficial effects on skin health, including antioxidant activity, maintenance of skin barrier function, wound healing, anti-inflammatory properties, and antimicrobial properties. Sugars, including polysaccharides, are notably abundant in both juniper and chamomile extracts [8,9,10]. They are rich in uronic acid and have demonstrated noteworthy moisturizing and hydrating properties [20,21,22]. These compounds act as humectants, effectively attracting and retaining water within the skin, thereby enhancing skin elasticity and promoting a supple complexion. Recent attention has been focused on the application of natural polysaccharides in diverse fields, including wound healing, anti-aging, and skin whitening, owing to their promising outcomes [23,24]. Apigenin 7-O-glucoside, quinic acid, coumarin derivatives, and ferulic acid glucosides are present in the extract combination and originate mainly from the chamomile extract (701), and their health-promoting activities have been described before [8,9,10,25,26,27,28]. These compounds effectively neutralize free radicals [29], mitigating oxidative stress and reducing signs of skin aging [30]. Apigenin and its derivatives, when topically applied, exhibit downregulation of inflammatory mediators, reducing inflammation in atopic skin [31,32,33]. It is noted that apigenin and chlorogenic acid contribute to enhancing skin barrier function [34,35]. Chlorogenic acid additionally demonstrates antioxidative capabilities, photoprotective effects, and modulation of skin collagen synthesis [36]. Ferulic acid [37], along with its derivatives, significantly contributes to anti-inflammatory and photoprotective actions and is widely used in cosmetic products [38,39]. The low phototoxicity and even slight photoprotective effect observed in our study might also be attributed to the presence of ferulic acid in the combination. Ferulic acid also inhibits tyrosinase activity and melanocyte proliferation, making it a common ingredient in skin-lightening cosmetic products [40]. Coumarin derivatives are known for their antimicrobial and anti-inflammatory properties [9,11,41]. Certain derivatives of coumarin have been recognized for their ability to inhibit tyrosinase, indicating their potential use in controlling skin pigmentation [42]. Moreover, coumarin has a high skin penetration rate, reaching up to 60% or even 100% depending on the vehicle used [43], thus making it available in dermal and subdermal layers. The JC extract supplements the combination with catechins, epigallocatechins, and gallocatechin, which possess antioxidant and antimicrobial properties, aiding in combating acne-causing bacteria and promoting collagen synthesis [17,21]. The high content of these compounds distinguishes JC extract from *M. recutita* extract (701), and the combination of both extracts increases the variety of catechins in the mixture, potentially leading to higher bioactivity. Importantly for cosmetic applications, these compounds are known to inhibit matrix-metalloproteinases [44]. The presence of catechins in products makes them more stable, bolsters protection against UV rays, and offers anti-aging effects [45,46]. Several flavonoids, including catechins, have previously demonstrated stimulating activities on skin cells [47,48]. Some of these compounds are known for their ability to modulate keratinocyte apoptosis and proliferation [49]. These findings, combined with the observed slight proliferation-stimulating effect, lead us to speculate that epigallocatechins and apigenin 7-O-glucoside specifically might be responsible for skin keratinocyte proliferation stimulating effect in the presence of JC extract and the combination. Thujaplicin derivatives are present in JC and the combination. These compounds exhibit diverse bioactivities, including antimicrobial and antifungal [50,51], antioxidative, and melanin synthesis-inhibiting properties [52,53,54,55]. Diterpenoid alkaloids, dihydroajaconine I and II, are present in JC. These compounds are still being studied for their biological effects, but they potentially possess anti-inflammatory activity [56]. Despite variations in compound concentrations, the observed differences in antioxidant activity raise discussion regarding the specific interactions and synergies between compounds within these extracts. When assessing the combined extract (JC + 701, 1:2) at varying dilutions (ranging from 1% to 25%) in water, there was a notable increase in substantial antioxidant activity. This suggests a potential synergistic interplay between compounds derived from both extracts, contributing to enhanced antioxidant capabilities, which corroborates previous observations [57,58]. Although the total content of flavonoids and phenolic compounds is lower in JC than in 701 and the combination, the high DPPH scavenging activity can be explained by the presence of specific compounds known for their high antiradical activity (e.g., gallocatechin, epigallocatechin, thujaplicin derivatives). Special emphasis should be placed on beta-thujaplicin derivatives, which are specific to the *Cupressaceae* plant family, as these compounds possess strong antioxidant activity [59,60]. The combination was superior regarding antioxidative activity in the intracellular ROS quantification assay, and the effect differed from that observed in the simpler DPPH scavenging test. This disparity could be attributed to the compounds’ capability not only to directly scavenge free radicals but also to stimulate intracellular stress response pathways. Such effects have been described in cell types other than skin cells before [61]. It was observed that when the concentration was increased (0.5% *v*/*v*), the intracellular ROS scavenging activity decreased. This is in line with previous publications, where it has been documented that several flavonoids and phenols exhibit dual anti- and pro-oxidative properties. The emergence of pro-oxidative activity occurs in both in vitro and in vivo biological systems when compounds interact with cellular proteins, catalysts, and the cellular antioxidative system, a phenomenon that might be concentration-dependent [62,63]. While phenolic compounds exhibit varying effects on collagen synthesis and their exact mechanisms are under study, there is increasing evidence that phenolics can reduce MMP-1 production in skin cells. Chlorogenic acid, present in the combination, is among the known plant secondary metabolites associated with the inhibition of MMP-1 synthesis and regulation of collagen production [48,64]. Catechin has also demonstrated MMP-1 inhibiting activity in other studies [65]. Flavonoids have different effects on skin extracellular matrix synthesis, with some of them inhibiting collagen synthesis and others promoting it [66]. It has been reported that apigenin glucosides promote collagen I synthesis in vitro [67,68]; however, in our study, *M. recutita* extract did not have an effect on collagen, while JC extract had a stimulating activity. β-thujaplicin is also known to have MMP-1-decreasing activity [69]. In our study, no changes in MMP-1 secretion were observed after incubation with β-thujaplicin glycosides containing JC extract, while the extract combination produced a slight decrease. This indicates the potential synergistic activities of various compounds in the mixture on MMP-1 production. Phytochemical screening and in vitro safety and bioactivity assays have revealed the potential benefits of tested extracts and their combination for cosmetic use. The results emphasize the importance of the testing of not only individual extracts but also their combinations to identify the bioactivities and target novel cosmetic ingredients for specific consumer demands. Comprehensive data on chemical composition, cytotoxicity, and potential activities on skin cells provide valuable preconditions for further cosmetic ingredient and product formulation developments and their assessments in dermatological studies.

## 4. Materials and Methods

### 4.1. Extracts and Development of the Combination

*Matricaria recutita* essential oil distillation process waste extract (designated 701) was produced by Field and Forest Ltd. using proprietary methodology. Alternative Plants Ltd. provided Juniperus communis callus culture extract (designated JC). In short, *J. communis* callus cultures were grown on solidified cultivation media, harvested, freeze-dried, and extracted with a mixture of glycerin and water solution using the company’s proprietary technology. A combination of two parts (*v*/*v*) 701 extract and one part JC extract was made and further characterized for its chemical composition, cytotoxicity, and biological activity.

### 4.2. Spectrophotometric Screening of Total Phenolics, Flavonoids, Tannins, Sugars, and Antioxidant Activity 

Following the guidelines previously described by Nakurte et al. [8], certain classes of secondary metabolites, including total phenols (TPC), total flavonoids (TFC), total tannins (TTC), and sugars, as well as antiradical activity (ARA/DPPH), were analyzed using an Epoch2 UV/Visible light Microplate Spectrophotometer (BioTek, Agilent, Waldbronn, Germany). The Folin–Ciocalteu test was used to estimate both TPC and TTC. 

In brief, for total phenolic content (TPC), 25 μL of the extract was mixed with 75 μL of H_2_O and 25 μL of Folin–Ciocalteu reagent (1:10) (Sigma-Aldrich, St. Louis, MO, USA) and allowed to stand for 6 min. Subsequently, 100 μL of a 7% Na_2_CO_3_ solution (Honeywell, Charlotte, NC, USA) was added and incubated for 90 min. The absorbance was read at 765 nm, and outcomes were indicated as mg gallic acid equivalents (Acros Organic, Geel, Belgium) per mL of extract material. 

For total tannin content (TTC), 50 μL of extract was combined with 50 μL H_2_O and 50 μL Folin–Ciocalteu reagent (1:1), followed by a 5 min resting period. Then, 100 μL of a 35% Na_2_CO_3_ solution was added and incubated for 30 min. The absorbance was measured at 700 nm, with the results presented as [mg] tannic acid equivalents (Alfa Aesar, Ward Hill, MA, USA) per mL of extract material. 

The aluminum chloride assay was used for total flavonoid content (TFC). A total of 20 μL of extract was mixed with 15 μL of 5% NaNO_2_ (Honeywell, Charlotte, USA) solution, followed by an incubation period of 5 min. Subsequently, 15 μL of a 10% AlCl_3_ solution (Acros Organics, Geel, Belgium) was added and allowed to stand for 6 min. Finally, 100 μL of a 1 M NaOH solution (Fisher Scientific, Hampton, VA, USA) was added and incubated for 15 min. The absorbance was measured at 510 nm, with results expressed both as mg apigenin equivalents (Rotichrom, Carl Roth GmbH, Karlsruhe, Germany) and as mg catechin (HWI Analytik GmbH, Rülzheim, Germany) per milliliter of extract material. 

Total sugar content was assessed using the Phenol–Sulfuric Acid Colorimetric Method. Additionally, 50 μL of extract was mixed with 150 μL of H_2_SO_4_ (Fisher Scientific, Hampton, VA, USA) and 30 μL of 5% phenol reagent (Alfa Aesar, Ward Hill, USA). The mixture was heated in an oven at 90 °C for 5 min, cooled, and the absorbance was read at 490 nm. Outcomes were indicated as mg glucose equivalents (Fisher Scientific, Hampton, USA) per milliliter of extract material. 

Moreover, the DPPH quenching activity assay determined the antioxidant activity of the extracts. A total of 20 μL of extract was mixed with 180 μL of 150 μM DPPH reagent (Alfa Aesar, Ward Hill, USA). The plate was kept in the dark at room temperature for 60 min, and absorbance was measured at 517 nm, expressed as μg Trolox equivalents (Acros Organics, Geel, Belgium) per milliliters of extract material. IC50 values, representing the concentration at which DPPH absorbance decreased by 50%, were determined by testing various extract concentrations. All calculated values were presented as the mean of three replicates ±SD.

### 4.3. Phytochemical Characterization of Extracts Using LC-qTOF-MS

The samples underwent filtration using a 0.45 μm RC (regenerated cellulose) filter before introduction into the chromatographic system. The separation method adhered to the procedure outlined by Nakurte et al. [8]. An Agilent 1290 Infinity II series high-performance liquid chromatography (HPLC) system coupled with an Agilent 6530 quadrupole time-of-flight mass spectrometry (qTOF MS) system (Agilent Technologies, Deutschland GmbH, Waldbronn, Germany) was utilized to analyze the obtained extracts. A Zorbax Eclipse Plus C18 Rapid Resolution HD column (Agilent Technologies, Deutschland GmbH, Waldbronn, Germany) (2.1 × 150 mm with a particle size of 1.8 μm) was employed, operating at a flow rate of 0.3 mL min^−1^, while maintaining the column oven at 50 °C. Injection of 1 μL of the sample included a 30 s needle wash using 70% methanol (Fisher Scientific, Hampton, USA). The mobile phase consisted of solvent A (0.1% formic acid (Fisher Scientific, Hampton, USA) in deionized water) and solvent B (0.1% formic acid in acetonitrile (Fisher Scientific, Hampton, USA). The gradient elution program comprised varying proportions of solvent B: initial 2% B, 0–2 min 2% B, 2–10 min 40% B, 10–20 min 80% B, 20–27 min 95% B, 27–40 min 95% B, and 40–42 min 1% B.

Operating parameters for the mass spectrometer included a fragmentation set at 70 V, gas temperature at 325 °C, a drying gas flow of 10 L min^−1^, nebulizer at 20 pounds per square inch (psi), sheath gas temperature at 400 °C, and sheath gas flow at 12 L min^−1^. Electrospray ionization (ESI) served as the source in positive mode, recording mass spectra within the *m*/*z* range of 50 to 2000. Internal reference masses of 121.050873 *m*/*z* and 922.009798 *m*/*z* (G1969-85001 ESI-TOF Reference Mass Solution Kit, Agilent Technologies & Supelco, Waldbronn, Germany) were employed for all sample analyses. Data from LCMS were analyzed using the Agilent MassHunter Qualitative Analysis 10.0 data acquisition software. Compounds were identified utilizing the Agilent MassHunter METLIN Metabolomics Database and the LipidMaps Database. Targeted individual standards were prepared for both individual compounds and compound classes’ quantification purposes. Content of quinic acids and derivatives were quantified by external calibration using chlorogenic acid (Acros Organics, Geel, Belgium) standard curve (0.1–100 µg mL^−1^), phenolic glycosides were quantified using ferulic acid (Acros Organics, Geel, Belgium) standard curve (1–100 µg mL^−1^), flavonoids were quantified using apigenin (Rotichrom, Carl Roth GmbH, Karlsruhe, Germany) standard curve (0.1–100 µg mL^−1^), coumarin derivatives were quantified using coumarin (Alfa Aesar, Ward Hill, USA) standard curve (0.1–100 µg mL^−1^), fatty acyls were quantified using valeric acid (Sigma-Aldrich, St. Louis, USA) standard curve (0.1–100 µg mL^−1^), epigallocatechins were quantified using catechin hydrate (HWI Analytik GmbH, Rülzheim, Germany) standard curve (0.1–100 µg mL^−1^), tropones were quantified using β-thujaplicin (Sigma-Aldrich, St. Louis, USA) standard curve (0.1–100 µg mL^−1^), diterpenoid alkaloids were quantified using senkirkine (PhytoLab, Vestenbergsgreuth, Germany) standard curve (0.05–50 µg mL^−1^), and amino acid and their derivative quantification were performed using leucine (Fisher Scientific, Hampton, USA) standard curve (0.1–50 µg mL^−1^), respectively. The analyses were performed in triplicate, and the presented results are means with a standard deviation (±SD).

### 4.4. Cytotoxicity

The in vitro cytotoxicity testing methodology was performed according to the method outlined in OECD Guidance document 129 [70]. BALB/3T3 cells (American Type Culture Collection (ATCC), Manassas, VA, USA) were plated in 96-well microplates at a concentration of 4 × 10^3^ cells per well. These cells were cultured in 100 μL of S10 medium (Dulbecco’s modified Eagle’s medium (DMEM) Sigma (Irvine, UK) supplemented with 1% penicillin (100 U mL^−1^)–streptomycin (100 μg mL^−1^) (P/S) (Sigma, St. Louis, MO, USA) and 10% calf serum (CS) (Sigma, St. Louis, MO, USA)) and left to incubate overnight at 37 °C with 5% CO_2_. Following this, the cells were washed with phosphate-buffered saline (PBS) (Sigma, Irvine, UK), and 100 μL of S5 medium (DMEM medium supplemented with 1% P/S and 5% CS) combined with the extract mix was introduced to the cells. Additionally, wells were prepared with the vehicle (appropriate solvent), S5 medium, and sodium dodecyl sulfate (SDS) (Sigma, St. Louis, USA) in S5 medium as controls. After 48 h of incubation at 37 °C with 5% CO_2_, the cells were rinsed with PBS, and 250 μL of 25 μg mL^−1^ Neutral Red dye solution (Sigma, Irvine, UK) in S5 medium was added to all wells. The plate was incubated for 3 h at 37 °C with 5% CO_2_, after which the cells were rinsed with PBS. Then, 100 μL of NR desorb solution (50% ethanol, 1% glacial acetic acid (Sigma, Irvine, UK), 49% water) was added to all wells. The plate was covered, placed in a microplate shaker for 20 to 45 min, and subsequently removed 5 to 10 min before measuring the absorbance at 540 nm using a Tecan M200 Infinite Pro microplate reader (Tecan, Mannedorf, Switzerland). Cell viability was determined as a percentage relative to the media control value, and IC50 values were also calculated.

### 4.5. Phototoxicity

The in vitro phototoxicity procedure was an adapted form of the method outlined in OECD Test Guideline 432 (TG 432) [71]. HaCaT keratinocytes (Cell Lines Services, Eppelheim, Germany) were treated with extracts in 96-well plates for one hour, then exposed to UVA light (5 J cm^−2^) utilizing UVACUBE 400 (Honle UV Technology, Gilching, Germany). Concurrently, cells were subjected to the extracts in darkness and evaluated under equivalent conditions. The assessment of Neutral Red dye uptake (NRU) was conducted after 24 h, following the procedure detailed in Section 4.4, titled “Cytotoxicity.”

### 4.6. Proliferation Analysis

Cell growth assessment was performed utilizing the Cell-IQ real-time cell monitoring platform (Chip-Man Technologies, Tampere, Finland) according to methodology adapted from the method described by Wang et al. [72]. Then, 10^4^ HaCaT keratinocytes were seeded into individual wells of a 24-well cell culture plate containing S10 medium (DMEM supplemented with 1% penicillin (100 U mL^−1^)–streptomycin (100 μg mL^−1^) (P/S) and 10% fetal bovine serum (FBS, Sigma, St. Louis, MO, USA)). These cells were cultivated at 37 °C with 5% CO_2_, enabling adhesion to the surface over a 4 h duration. Subsequently, fresh media infused with the compound mixture were introduced, instigating the examination of cell proliferation using the Cell-IQ system. Four fields of view were selected for monitoring and subsequent analysis within each well. 

### 4.7. Flow Cytometric Quantification of Reactive Oxygen Species (ROS) in HaCaT Keratinocytes

The CellROX™ Green flow cytometry assay kit (Thermo Fisher Scientific, C10492, Waltham, MA, USA) was employed following the recommended protocol with minor modifications. Briefly, HaCaT cells (immortalized human skin keratinocytes) (Cell Lines Services, Eppelheim, Germany) were cultured in media (10% FBS/DMEM) within T75 cultivation flasks. Upon reaching confluency, cells were detached using trypsin (Sigma, St. Louis, USA) and suspended at a concentration of 3 × 10^5^ cells per 100 μL in media containing the combination, followed by a 1 h incubation at +37 °C with 5% CO_2_. As a control, an antioxidant sample was incubated at +37 °C with 5% CO_2_ with N-acetyl cysteine (NAC) (500 μM) (Invitrogen, Eugene, OR, USA) and a solvent control for 1 h before the induction of ROS. UVA radiation of 5 J cm^−2^ in 70 μL PBS within open 1.5 mL Eppendorf tubes was utilized for ROS induction. Subsequently, cells were left to incubate for an additional hour in 100 μL PBS/10% FBS.

Staining was performed using CellROX™ Green (500 nM) (Invitrogen, Oregon, USA) for 40 min and SYTOX™ Red Dead Cell Stain (5 nM) (Invitrogen, Oregon, USA) for 10 min in the dark. Samples were analyzed using the Amnis FlowSight imaging flow cytometer (EMD Millipore, Washington, DC, USA), acquiring 10,000 events per sample. In the final analysis, only focused singlets and SYTOX™ Red Dead Cell Stain-negative cells were included. The Median Fluorescence Intensity (MFI) served as a reference for evaluating the antioxidative properties of the analyzed sample treatment; lower MFI indicates better scavenging properties and protection from ROS-induced damage to cells.

### 4.8. Secretion of Pro-Collagen I and MMP-1

Human primary dermal fibroblasts HDFa (ATCC, Manassas, VA, USA) were seeded in a 24-well plate at a density of 1.5 × 10^4^ cells per well in S10 cultivation media (DMEM supplemented with 1% penicillin (100 U mL^−1^)–streptomycin (100 μg mL^−1^) (P/S) and 10% FBS). The cells were incubated for 24 h at +37 °C with 5% CO_2_ to allow them to attach and initiate proliferation. Fresh media containing a combination of extracts were added, and the cells were cultivated at +37 °C with 5% CO_2_. Media samples were collected for analysis after 24 and 72 h of incubation with the combination. The secretion of pro-collagen I and MMP-1 in the cultivation media was quantified using ELISA immunoassays employing R&D Systems DuoSet Human pro-collagen I alpha 1 and MMP-1 ELISA kits (R&D Systems, Minneapolis, MN, USA), following the manufacturer’s recommendations. Standard dilutions of pro-collagen I (R&D Systems Minneapolis, USA) and MMP-1 (R&D Systems Minneapolis, USA) were utilized to generate the standard curve and calculate the concentrations of both analytes in the cultivation media.

### 4.9. Statistical Analysis

Data were analyzed using GraphPad Prism 9 software (GraphPad, La Jolla, CA, USA). One-way ANOVA test was used to analyze the impact of origin of the extract on the TPC, TFC, total sugar content, ARA, and ROS. Two-way ANOVA test was used to analyze pro-collagen I alpha 1 and MMP-1 secretion data.

## 5. Conclusions

The development and characterization of the extract combination in this study provide compelling evidence of the advantages offered by multicomponent cosmetic ingredients and emphasize the need to evaluate not only individual extracts but also their combinations before incorporation into cosmetic products. The utilization of two different plant sources results in a rich blend of compounds in the combination. The blend of extracts derived from juniper in vitro callus biomass and chamomile processing waste demonstrates low cytotoxicity and no phototoxicity. Its efficacy in significantly reducing in vitro accumulation of free radicals in skin keratinocytes was proven. The combination did not show superiority compared to the JC extract in the cell proliferation test and pro-collagen production assay. At the same time, it should be emphasized that JC’s high collagen synthesis-promoting activity also contributes to the combination’s positive effect on collagen production. Compared to individual extracts, the combination slightly impedes the production of collagen-degrading metalloproteinase MMP-1. These findings underscore the considerable potential of this combination for further development as a skin-protecting, anti-aging cosmetic ingredient. Furthermore, the high sustainability of both components within the combination adds an extra layer of attractiveness for the industry. Safety and efficacy results from the in vitro assays provide valuable preconditions to move forward with testing of the combination in dermatological tests.

## Figures and Tables

**Table 1:** Content of total phenolics (TPC), flavonoids (TFC), and tannins (TTC) and sugars in *M. recutita* processing waste extract (701), *J. communis* callus culture extract (JC), and combination extract (JC + 701, 1:2). Values represent the mean of three determinations ± SD.

Extract	TPC ^a^, GAE mg mL^−1^	TFC ^b^, APE mg mL^−1^	TFC ^c^, CAE mg mL^−1^	TTC ^d^, TAE mg mL^−1^	Sugars ^e^, GLE mg mL^−1^
701	1.83 * ± 0.01	1.79 * ± 0.03	1.54 * ± 0.01	2.21 * ± 0.02	78.88 * ± 0.04
JC	0.35 * ± 0.01	0.19 * ± 0.01	0.48 * ± 0.01	2.64 * ± 0.01	5.60 * ± 0.01
JC + 701	1.57 * ± 0.01	0.79 * ± 0.04	1.33 * ± 0.01	1.45 * ± 0.02	24.22 * ± 0.03

^a^ Total phenolic content is expressed as gallic acid equivalents per milliliter of extract (mg GAE mL^−1^). ^b^ Total flavonoid content is expressed as the apigenin equivalent per milliliter of extract (mg APE mL^−1^). ^c^ Total flavonoid content is expressed as the catechin equivalent per milliliter of extract (mg CAE mL^−1^). ^d^ Total tannin content is expressed as the tannic acid equivalents per milliliter of extract (mg TAE mL^−1^). ^e^ Total sugar content is expressed as the glucose equivalents per milliliter of extract (mg GLE mL^−1^). The ANOVA analysis showed significant differences, represented by asterisk * (*p* < 0.001), between the class of compounds and the origin of the extract.

**Table 2:** 2,2-diphenyl-1-picrylhydrazyl (DPPH) free radical scavenging activity of *M. recutita* processing waste extract (701), *J. communis* callus culture extract (JC), and combination extract (JC + 701, 1:2). Values represent the mean of three determinations ± SD.

Extract	ARA ^a^, TE µg mL^−1^	DPPH ^b^ Quenched, %	IC 50, mg mL^−1^
701	22.1 * ± 0.01	57.6 * ± 0.13	0.97 * ± 0.05
JC	91.3 * ± 0.02	62.0 * ± 0.15	2.00 * ± 0.06
JC + 701 (1%) ^c^	16.9 * ± 0.02	13.7 * ± 0.02	1.91 * ± 0.05
JC + 701 (5%) ^c^	50.5 * ± 0.03	35.5 * ± 0.02	
JC + 701 (10%) ^c^	90.8 * ± 0.04	61.6 * ± 0.06	
JC + 701 (25%) ^c^	110.9 * ± 0.06	74.6 * ± 0.05	

^a^ ARA radical scavenging activity is expressed as the trolox equivalents per milliliter of extract (µg TE mL^−1^). ^b^ 2,2-diphenyl-1-picrylhydrazyl (DPPH) radical scavenging activity of extracts expressed in %. ^c^ Dilution of extract concentration in water. The ANOVA analysis showed significant differences, represented by asterisk (*p* < 0.001), between the origin of the extract.

**Table 3:** Quantification of dominant phytocomponents in *M. recutita* processing by-product extract and *J. communis* callus culture extract combination (JC + 701, 1:2) with indication of compound presence in individual extracts. Values represent the mean of three determinations ± SD.

Number	Proposed Compound	Formula	JC + 701, µg mL^−1^	Source
Amino Acids and Derivatives ^a^, µg mL^−1^				
1	Homostachydrine	C_8_H_15_NO_2_	16.98 ± 0.19	701
2	N-(1-deoxy-D-fructos-1-yl)-L-Serine	C_9_H_17_NO_8_	14.52 ± 0.33	JC; 701
3	Isoleucine	C_6_H_13_NO_2_	11.07 ± 0.09	JC; 701
4	Leucine	C_6_H_13_NO_2_	20.20 ± 0.11	JC; 701
5	Tyrosine	C_9_H_11_NO_3_	3.49 ± 0.08	JC; 701
6	Phenylalanine	C_9_H_11_NO_2_	6.74 ± 0.09	JC; 701
7	Tyrosyl-Tyrosine	C_18_H_20_N_2_O_5_	2.54 ± 0.04	JC; 701
	Total	75.53 ± 0.45		
Flavonoids/Epigallocatechins ^b^, µg mL^−1^				
8	(−)-Epigallocatechin	C_15_H_14_O_7_	2.72 ± 0.09	JC; 701
9	Epicatechin-(4β⟶8)-gallocatechin	C_30_H_26_O_13_	1.59 ± 0.06	JC
10	(+)-Gallocatechin	C_15_H_14_O_7_	1.00 ± 0.06	JC
11	(+)-Catechin	C_15_H_14_O_6_	0.95 ± 0.05	JC
	Total	6.26 ± 0.12		
Flavonoids ^c^, µg mL^−1^				
12	Apigenin 7-O-glucoside	C_21_H_20_O_10_	14.39 ± 0.23	701
	Total	14.39 ± 0.23		
Quinic acids and derivatives ^d^, µg mL^−1^				
13	Neochlorogenic acid	C_16_H_18_O_9_	15.41 ± 0.28	701
14	Chlorogenic acid	C_16_H_18_O_9_	36.29 ± 0.34	701
15	Cryptochlorogenic acid	C_16_H_18_O_9_	18.78 ± 0.09	701
	Total	70.48 ± 0.63		
Coumarins and derivatives ^e^, µg mL^−1^				
16	Skimmin	C_15_H_16_O_8_	8.28 ± 0.08	701
17	Herniarin	C_10_H_8_O_3_	13.71 ± 0.11	701
	Total	21.99 ± 0.10		
Phenolic glycosides ^f^, µg mL^−1^				
18	Ferulic acid O-glucoside I	C_16_H_20_O_9_	880.8 ± 0.67	701
19	Ferulic acid O-glucoside II	C_16_H_20_O_9_	223.4 ± 0.25	701
	Total	1104.21 ± 0.95		
Tropones ^g^, µg mL^−1^				
20	β-Thujaplicin glycoside I	C_16_H_22_O_7_	0.97 ± 0.02	JC
21	β-Thujaplicin glycoside II	C_16_H_22_O_7_	1.04 ± 0.02	JC
	Total	2.01 ± 0.02		
Diterpenoid alkaloids ^i^, µg mL^−1^				
22	Dihydroajaconine I	C_22_H_35_NO_3_	0.27 ± 0.01	JC
23	Dihydroajaconine II	C_22_H_35_NO_3_	0.03 ± 0.00	JC
	Total	0.30 ± 0.01		
Fatty Acyls ^j^, µg mL^−1^				
24	3-Hydroxyphenyl-valeric acid	C_11_H_14_O_3_	14.30 ± 0.03	701
25	Isobutyl-2-methylbutyrate	C_9_H_18_O_2_	28.20 ± 0.07	701
	Total	42.50 ± 0.15		

^a^ Leucine equivalents. ^b^ Catechin hydrate equivalents. ^c^ Apigenin equivalents. ^d^ Chlorogenic acid equivalents. ^e^ Coumarin equivalents. ^f^ Ferulic acid equivalents. ^g^ β-Thujaplicin equivalents. ^i^ Senkirkine equivalents. ^j^ Valeric acid equivalents.

## Data Availability

Data are contained within the article.
